# AptaShield: A Universal Signal-Transduction System for Fast and High-Throughput Optical Molecular Biosensing

**DOI:** 10.1021/acssensors.3c02762

**Published:** 2024-04-15

**Authors:** Miguel António Dias Neves, Inês Mendes Pinto

**Affiliations:** † Institute for Research and Innovation in Health (i3S), University of Porto, 4200-135 Porto, Portugal; ‡ Molecular and Analytical Medicine Laboratory, Department of Biomedicine, Faculty of Medicine, University of Porto, 4200-319 Porto, Portugal

**Keywords:** Aptamers, Universal transducer, High-throughput, Solution-phase biosensor, Optical detection, Molecular profiling

## Abstract

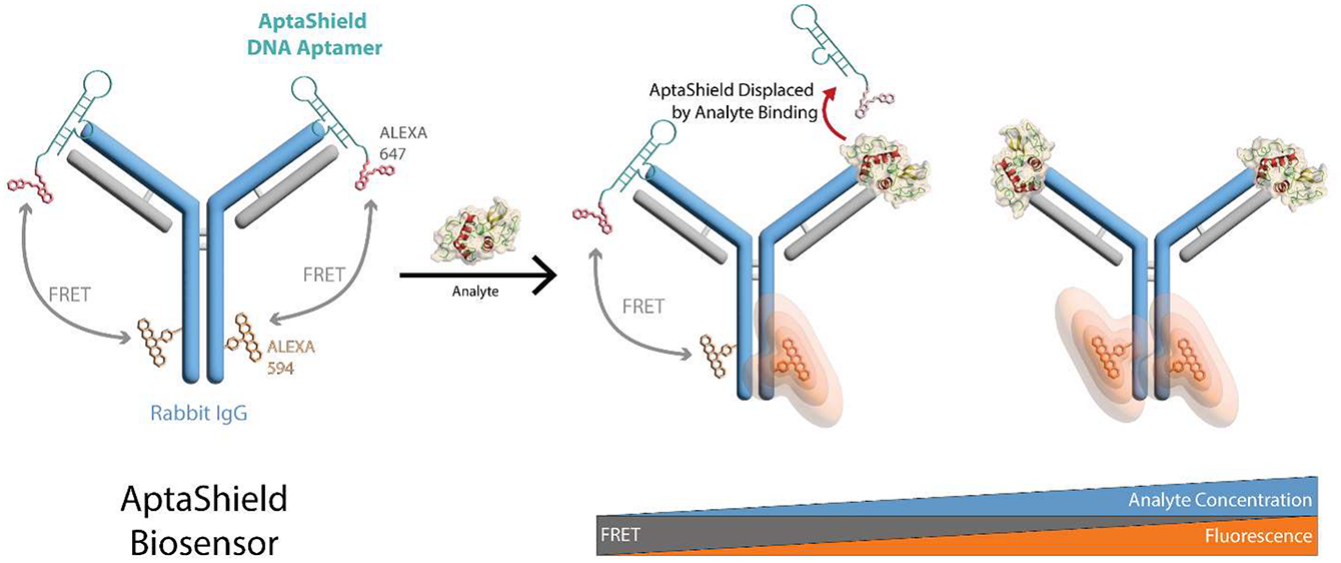

Biosensing technologies are often described to provide facile, sensitive, and minimally to noninvasive detection of molecular analytes across diverse scientific, environmental, and clinical diagnostic disciplines. However, commercialization has been very limited mostly due to the difficulty of biosensor reconfiguration for different analyte(s) and limited high-throughput capabilities. The immobilization of different biomolecular probes (e.g., antibodies, peptides, and aptamers) requires the sensor surface chemistry to be tailored to provide optimal probe coupling, orientation, and passivation and prevent nonspecific interactions. To overcome these challenges, here we report the development of a solution-phase biosensor consisting of an engineered aptamer, the AptaShield, capable of universally binding to any antigen recognition site (Fab’) of fluorescently labeled immunoglobulins (IgG) produced in rabbits. The resulting AptaShield biosensor relies on a low affinity dynamic equilibrium between the fluorescently tagged aptamer and IgG to generate a specific Förster resonance energy transfer (FRET) signal. As the analyte binds to the IgG, the AptaShield DNA aptamer–IgG complex dissociates, leading to an analyte concentration-dependent decrease of the FRET signal. The biosensor demonstrates high selectivity, specificity, and reproducibility for analyte quantification in different biological fluids (e.g., urine and blood serum) in a one-step and low sample volume (0.5–6.25 μL) format. The AptaShield provides a universal signal transduction mechanism as it can be coupled to different rabbit antibodies without the need for aptamer modification, therefore representing a robust high-throughput solution-phase technology suitable for point-of-care applications, overcoming the current limitations of gold standard enzyme-linked immunosorbent assays (ELISA) for molecular profiling.

Biosensors are analytical devices combining a biological molecular recognition component with a physical-chemical component to produce a measurable analyte concentration-dependent signal (e.g., electrochemical, optical, mass), schematically outlined in Figure 1a.^1,2^ The market landscape for biosensors is promising, given their potential for fast, remote, and easy molecular detection potentially suitable for various point-of-care diagnostics and environmental sensing applications.^3,4^ Despite the extensive research and development in sensor technology, the translation of these advancements into commercial products has been slow, leading to a notable gap between research and market availability.^5,6^ This discrepancy can be attributed, at least, to three fundamental requirements limiting the reconfiguration capacity of current biosensing technologies: (1) physical or chemical immobilization of the molecular recognition probe onto the biosensor surface; (2) detection of different targets often demands reengineering of the signal transduction scheme;^7,8^ and (3) surface chemistry tailoring to prevent nonspecific interactions between the molecular recognition probe and the sensor surface and nonspecific adsorption of sample matrix components, potentially impairing the analytical performance of the sensor.^9−12^ In recent years, there have been efforts to utilize nanomaterial-based transducers, such as metallic nanoparticles, to leverage signal-transduction systems from bulk solid to a solution-like phase for optical, spectroscopic, or electrochemical analyte detection.^13−15^ Despite these efforts, challenges persist in fully overcoming all associated and previously listed issues.

**Figure 1:**
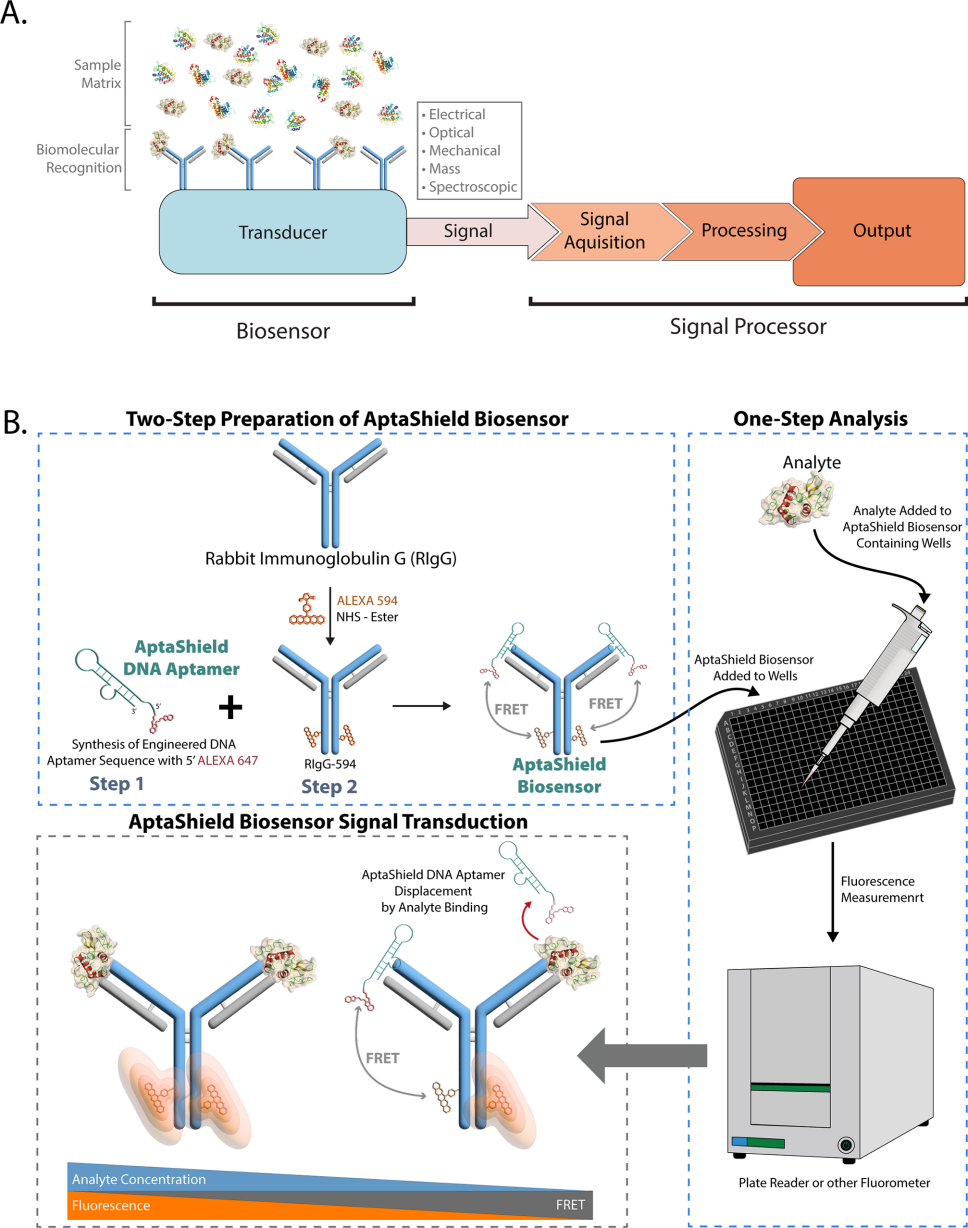
(a) Block diagram of a conventional biosensor and (b) schematic representation of the fabrication and detection mechanism of the AptaShield biosensor.

In this work, we introduce AptaShield (Figure 1b), a versatile aptamer-based signal transduction technology capable of transforming any rabbit immunoglobulin G (RAH) into a solution-based optical biosensor, enabling streamlined high-throughput analysis in a single step (Figure 1b, right panel). Importantly, the rabbit is a primary source of antibodies used in molecular diagnostics and therapeutics.

The AptaShield biosensor relies on a low affinity dynamic equilibrium between a fluorescently labeled AptaShield DNA aptamer and RAH which generates a Förster resonance energy transfer (FRET) signal. In this system, when the analyte of interest is introduced, the AptaShield–RAH complex dissociates due to a higher binding affinity between the RAH and the analyte, leading to a FRET signal decay, correlated with the analyte concentration. As a proof-of-concept, the AptaShield biosensor was tested for the one-step quantitative detection of the analyte, human Fc fragment, in a high-throughput (384-well plate) format with low sample volume requirements (0.5–6.25 μL). We show that the AptaShield biosensor has high selectivity, specificity, tunability, and reproducibility for analyte quantification in different biological fluids, such as urine and blood serum, highlighting its potential applicability for different biosensing applications.

The AptaShield corresponds to a truncated DNA aptamer (Table S1) whose sequence is derived from aptamers selected against a polyclonal pool of rabbit IgGs to bind to conserved motifs within the Fab fragment, sterically blocking antigen binding.^16^ Importantly, the Fab fragments of antibodies are composed of constant and variable domains. The variable domain includes complementarity-determining (CDR) and framework (FW) regions comprising ∼25% and ∼75% of the variable domain, respectively.^17−19^ The FWs are highly conserved regions within the variable domain that do not interact with the antigen. Instead, they are responsible for providing structural support for the CDRs. On the other hand, CDRs directly interact with the antigen and are responsible for achieving high-affinity binding to various epitopes, hence their high variability.^17,19,20^ Therefore, a polyclonal mixture consists of antibodies that bind to different epitopes/antigens and have different CDRs. Considering the overall antibody structure, the selected aptamer was designed to bind conserved FW regions, sterically hindering the CDRs. In this framework, the same aptamer provides a universal platform for binding to different IgGs (produced in rabbits) for detection of different antigens without any need of aptamer reengineering.

Binding of the AptaShield DNA aptamer to IgGs was evaluated using isothermal titration calorimetry (ITC) which allows for the label-free thermodynamic characterization of intermolecular interactions. Experiments were performed by titrating the AptaShield DNA aptamer into unlabeled purified rabbit IgGs from rabbit serum, rabbit antihuman monoclonal IgG (RAH), and mouse antihuman IgG. The resulting thermograms were corrected for the heat of dilution of the titrant and fit to a one set of sites binding model (Figure 2a–c). The determined ITC parameters are detailed in Table S2, and ITC heats of dilution are in Figure S1. The ITC data demonstrated that the AptaShield DNA aptamer selectively binds to the purified rabbit IgGs and RAH with an apparent dissociation constant of 890 ± 657 nM and 224 ± 92 nM, while no binding was observed with the mouse antihuman IgG.

**Figure 2:**
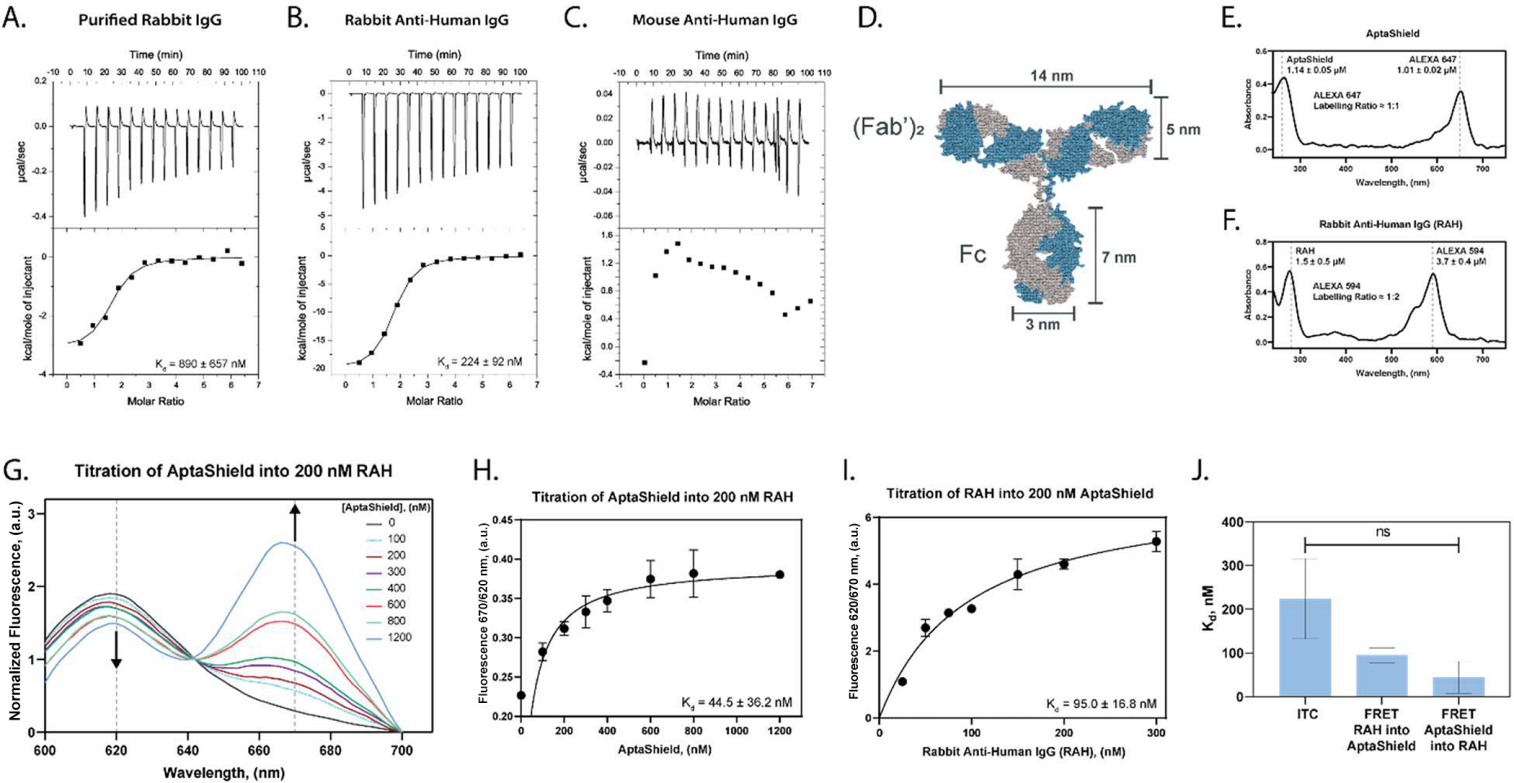
AptaShield DNA aptamer–IgG binding. Isothermal titration calorimetry (ITC) thermograms related to the titration between the AptaShield DNA aptamer and (a) purified IgGs from rabbit serum, (b) monoclonal rabbit antihuman IgG, and (c) monoclonal mouse antihuman IgG. Monitoring AptaShield DNA aptamer–rabbit IgG binding by FRET. (d) Depiction of the 3D structure of an IgG with corresponding dimensions. UV–vis spectra of (e) the ALEXA-647-labeled AptaShield DNA aptamer and (f) ALEXA-594-labeled rabbit antihuman IgG (RAH). The molar ratio of AptaShield DNA aptamer/RAH to fluorophore was determined using the relevant extinction coefficients (tabulated in Tables S1 and S2). The AptaShield DNA aptamer was found to be labeled at a 1:1 ratio, while the RAH was found to be labeled at a 2.3 (fluorophore):1 (RAH) ratio. (g) Fluorescence emission (Ex 550 nm) spectra of the titration of different concentrations of AptaShield DNA aptamer into 200 nM RAH. Binding curves related to the titration of (h) AptaShield DNA aptamer into 200 nM RAH and (i) RAH into 200 nM AptaShield DNA aptamer. (j) Statistical analysis of the AptaShield DNA aptamer–RAH dissociation constants (*K*_d_) determined from ITC and FRET.

Quantitative analyte detection by the AptaShield biosensor correlates with the FRET signal decay caused by dissociation of the AptaShield–rabbit IgG complex. For this purpose and considering the typical dimensions of an IgG (Figure 2d), a long-range FRET pair was selected: ALEXA-594 (donor – Em 590 nm, Ex 620 nm) and ALEXA-647 (acceptor – Em 650, Ex 670 nm), with a Förster distance (*R*_0_) of 8.5 nm. The AptaShield DNA aptamer sequence was engineered with a 5′-ALEXA 647, while the rabbit IgG was chemically labeled with ALEXA-594 succinimidyl (NHS) ester. The sensitivity of the AptaShield biosensor is dependent on the labeling efficiency of the Rabbit IgG, and the labeling reaction was found to have an efficiency of 2.3 mol of fluorophore per mol of RAH (Figure 2e and f). To evaluate the robustness of the FRET signal, different concentrations of AptaShield were titrated into 200 nM RAH-594 and the fluorescence emission spectra (Ex 550 nm) acquired using a Molecular Devices SpectraMax iD3 (Figure 2g). Additionally, fluorophore stability was assessed during a 10 min time frame, and no significant photobleaching was observed, as shown in Figure S2. The spectra (Figure 2g) clearly show that as the AptaShield DNA aptamer concentration increases the fluorescence intensity at 620 nm decreases (RAH-594) and increases at 670 nm, demonstrating a correlation between AptaShield DNA aptamer concentration in solution and the FRET signal increase. Ratiometric FRET analysis was used to determine the binding affinity of the interaction between the AptaShield DNA aptamer and RAH. In this context, two titrations were performed; AptaShield was titrated into RAH and RAH into AptaShield; and the fluorescence intensity ratio of the donor (RAH-594) and acceptor (AptaShield DNA aptamer-647) was normalized against the background signal of the acceptor and plotted against an AptaShield DNA aptamer or RAH concentration (Figure 2h and i). The corresponding curves were fit to a one set of sites binding model, and the apparent dissociation constants of the interaction were determined to be 44.5 ± 36.2 nM and 95.0 ± 16.8 nM, respectively. This analysis demonstrates that RAH labeling does not interfere with AptaShield DNA aptamer–RAH binding and that the intermolecular interactions can be monitored with high sensitivity using FRET. The binding affinities determined by FRET are not statistically different from those obtained from ITC, as shown in Figure 2J.

As a proof-of-concept, three AptaShield biosensors were constructed with varying stoichiometric ratios of the AptaShield DNA aptamer and 594-RAH, labeled as 150/50, 300/50, and 600/50 (Figure 3a). These ratios correspond to approximately 60%, 80%, and 90% of the RAH binding sites being occupied by the AptaShield DNA aptamer, based on the binding affinities determined in Figure 2h and i. For analyte detection, a purified human IgG Fc fragment was added to each well of a 384-well plate containing AptaShield biosensor and then the fluorescence intensity determined: excitation 550 nm, emission intensity donor (*I*_D_) 620 nm, and emission intensity acceptor (*I*_A_) 670 nm. The analytical performances of 150/50, 300/50, and 600/50 AptaShield biosensors were first determined in PBS buffer. Increasing concentrations (0.1–1000 nM) of human Fc fragment were added to each biosensor, and the FRET (*I*_A_/*I*_D_ subtracted with the background signal of the acceptor, AptaShield-647) was analyzed (Figures 3b and S3). The data were then transformed into FRET^–1^ (*I*_D_/*I*_A_) (Figure 3c) for calculation of the apparent dissociation constant (*K*_d Apparent_) and limit of detection (*C*_LoD_) (as described in the Supporting Information). The linear range, *K*_d Apparent_, and C_LoD_ of each AptaShield biosensor are described in Figure 4d. The *C*_LoD_s were determined to be 1.33 ± 0.33, 0.36 ± 0.18, and 0.05 ± 0.01 nM for the 150/50, 300/50, and 600/50 AptaShield biosensors, respectively, demonstrating that the sensitivity and linear range of the AptaShield biosensor can be tuned by adjusting the stoichiometry between the AptaShield DNA aptamer and RAH. The *K*_d Apparent_ of the different AptaShield biosensors decreases as the ratio of the AptaShield DNA aptamer is increased (Figures 3c and 4d) which may seem counterintuitive when compared to traditional IC_50_ measurements. However, in the case of the AptaShield biosensor, the lower *K*_d Apparent_ arises due to the high background acceptor signal (*I*_A_) that must be subtracted (Figure S4). The 300/50 ratio exhibits twice the background signal, and the 600/50 ratio exhibits three times the background signal of the 150/50 ratio. This indicates a lower signal-to-noise ratio as the amount of the AptaShield DNA aptamer is increased. This is illustrated in Figure 3b, where the initial FRET intensities (at the lowest human Fc concentrations) are lower in the AptaShield biosensors with a higher amount of aptamer, indicating the reduced signal-to-noise ratio as the aptamer is increased, ultimately causing the signal to saturate at lower human Fc concentrations.

**Figure 3:**
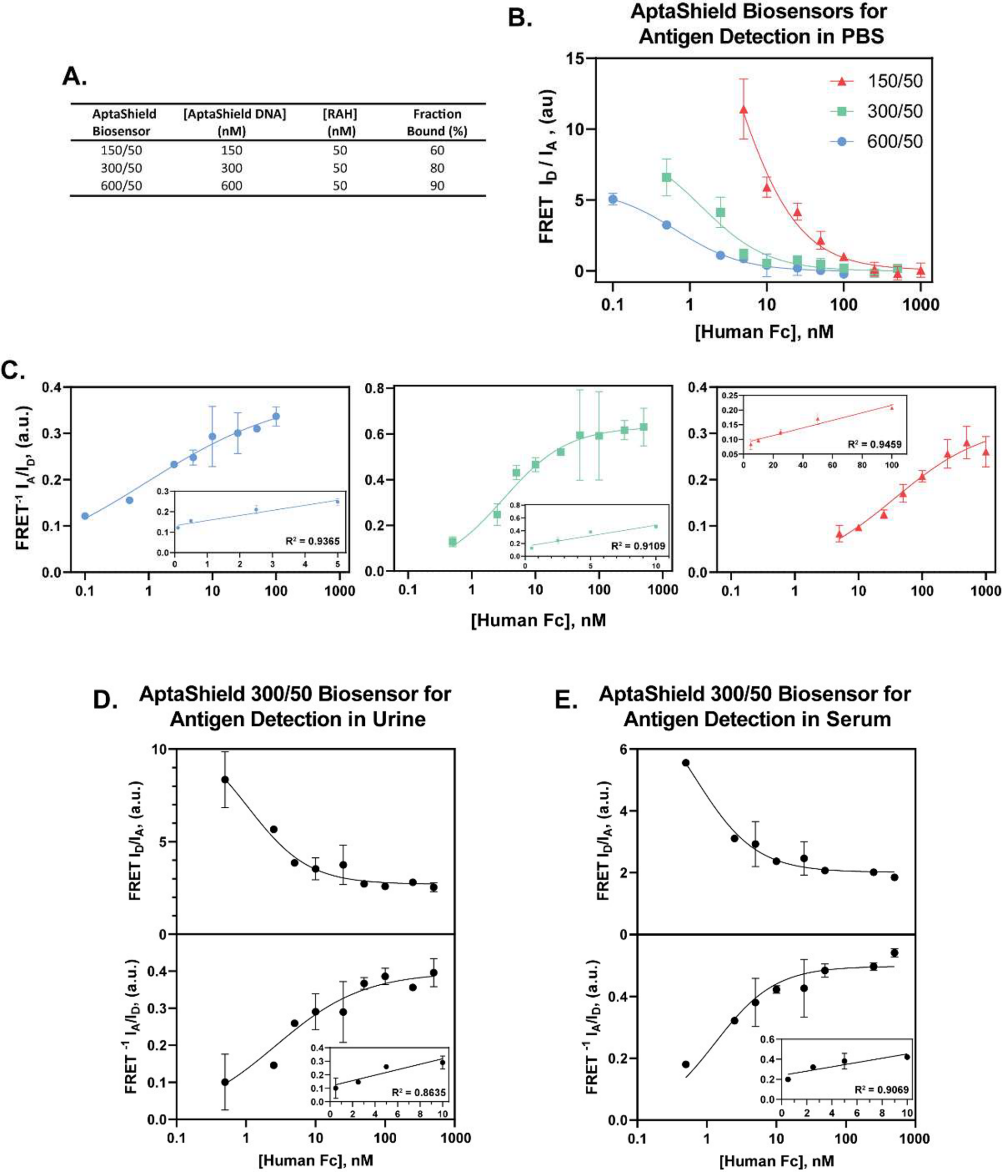
Analytical performance of the AptaShield biosensor. The proof-of-concept AptaShield biosensor was configured to detect purified human Fc fragment. (a) Different AptaShield biosensors were constructed by varying the ratio between the aptamer and RAH and (b) the baseline subtracted FRET (*I*_D_/*I*_A_) response curves of these different AptaShield biosensors to different concentrations of human Fc fragment. The nonbaseline corrected binding curves are depicted in Figure S3. (c) Inversion of the FRET response curves (FRET^–1^, *I*_A_/*I*_D_) for determination of the *K*_d Apparent_ and *C*_LoD_ of the different AptaShield biosensors. Linear regions of the curves are inset. FRET and FRET^–1^ response curves of 300/50 AptaShield biosensor to different concentrations of human Fc spiked (d) bovine urine and (e) horse serum. The values are presented as mean ± SEM from 3 to 5 replicas per condition. Statistical analysis was performed using two-tailed *t* test, and the symbol ** corresponds to *p* < 0.01.

**Figure 4:**
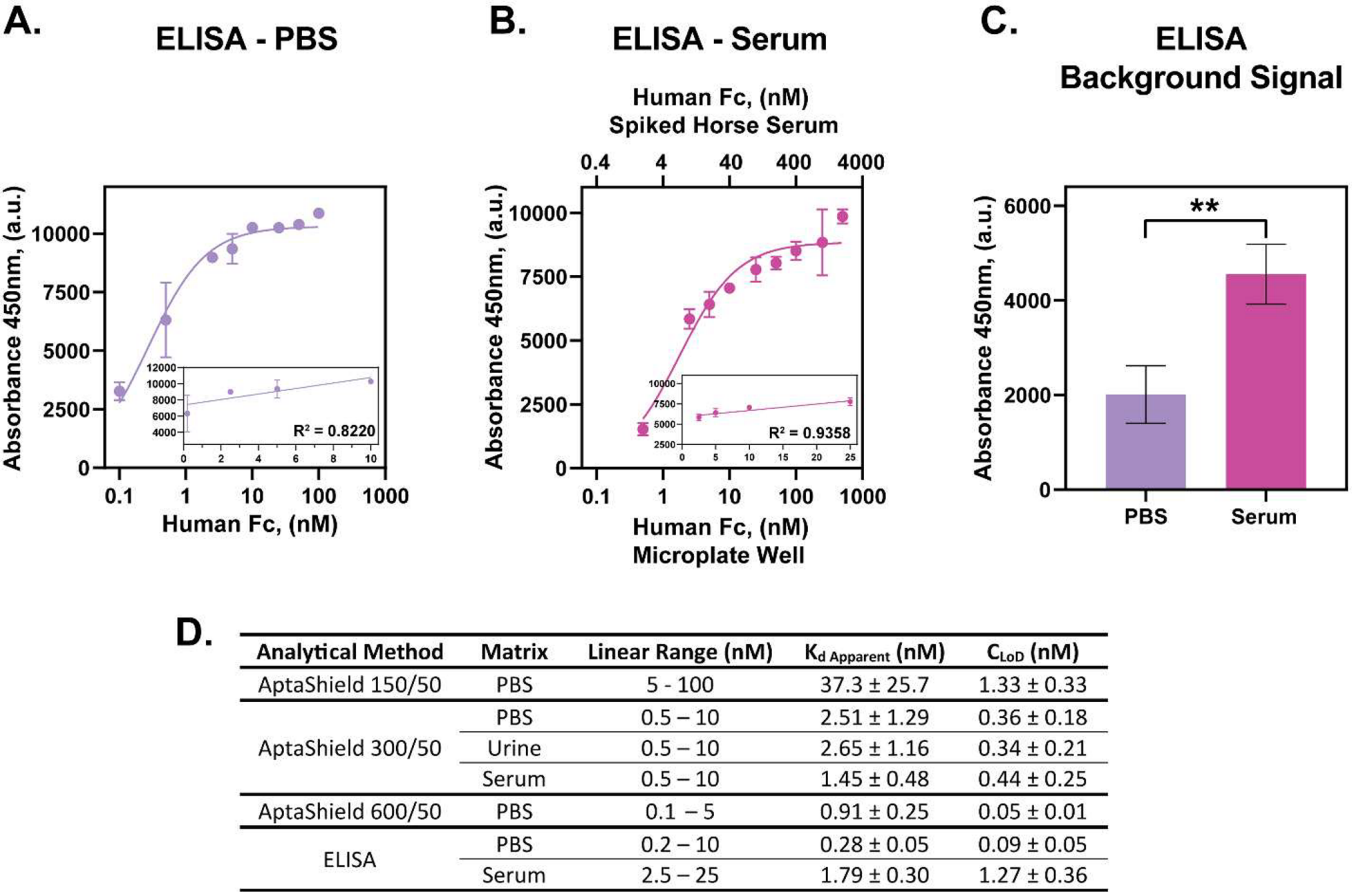
Comparison of the analytical performance of the AptaShield biosensor with ELISA. Comparative direct sandwich ELISA concentration response curve for the detection of human Fc in (a) PBS and (b) horse serum. Inset are the linear regions of the curves. (c) ELISA background signal of analyte free PBS and horse serum, which were subtracted from the values to produce the concentration response curves. (d) Multiparametric analysis of the AptaShield biosensor and ELISA performance for analyte detection in PBS, blood serum, and urine. The values are presented as mean ± SEM from 3 to 5 replicas per condition. Statistical analysis was performed using two-tailed *t* test, and the symbol ** corresponds to *p* < 0.01.

In this current solution-phase format, the AptaShield biosensor has significant potential in point-of-care and high-throughput applications. Within this framework, the AptaShield biosensor was adapted for the detection of human Fc in spiked horse blood serum and bovine urine samples. In this configuration, 6.25 μL of spiked blood serum or urine samples was added to the wells of a 384-well plate containing 18.75 μL of 300/50 AptaShield biosensor to a final sensor volume of 25 μL. The FRET and FRET^–1^ response curves to different concentrations of human Fc spiked urine and blood serum samples are depicted in Figure 3d and e and the analytical parameters in Figure 4d. In the urine and blood serum samples, the apparent *K*_d_ and the *C*_LoD_ were not statistically different when compared to PBS. The AptaShield biosensor was then compared against a “gold standard” direct sandwich enzyme-linked immunosorbent assay (ELISA) in both PBS and spiked blood serum. The concentration response curves of ELISA and analytical parameters are depicted in Figure 4a, b, and d. ELISA human Fc detection sensitivity in PBS was shown to be comparable to that of the 600/50 AptaBody biosensor but with an enlarged linear range. However, the analytical performance of ELISA in spiked serum samples was greatly reduced compared to PBS (CLoD of 0.09 ± 0.05 nM compared to 1.27 ± 0.36 nM). This phenomenon was not observed with the solution-phase AptaShield biosensor, highlighting the potential fouling of matrix components on the ELISA microplate surface, therefore preventing effective antibody binding.

The AptaShield biosensor is a first-of-its-kind aptamer-based signal transduction technology that can be combined with a rabbit IgG to create an AptaShield biosensor (Figure 1). We demonstrated that the AptaShield DNA aptamer binds with specificity toward rabbit IgGs (both monoclonal and a mixture of purified antibodies from rabbit serum) and does not bind mouse IgGs (Figure 2). The AptaShield biosensor was found to quantitatively detect human Fc in buffer, blood serum, and urine matrices with analytical performance comparable to ELISA assays and achieving limits of detection less than 1.5 nM (Figure 4d). Furthermore, the AptaShield biosensor operates in the solution phase, providing new means for in-solution detection with high reproducibility while minimizing the risk of fouling. Compatibility with the 384-well microplate format reinforces the integration of the biosensor with common automated liquid handling and a high-throughput screening apparatus and many ubiquitous fluorescence signal processing instruments ranging from a standard microplate plate reader, fluorimeter, to portable compact spectrophotometers using LED light sources (such as those from OceanOptics) suitable for various sensing applications in remote settings. In contrast to the AptaShield biosensor, ELISAs are time-consuming and multistep methodologies requiring systematic and individual chemical modifications of different antibodies to allow recognition of different antigens in a solid-phase platform.

Commonly reported biosensor technologies requires chemical immobilization and subsequent structural orientation of a biomolecular recognition probe on the sensor surface to facilitate the conversion of analyte–probe interactions into a measurable signal (Figure 1a).^1,2^ This challenges the reconfiguration capacity of the biosensor for the detection of different analytes, as the underlying surface chemistry must be tailored to accommodate different recognition probes. On this basis, we postulate that the limitations of biosensor reconfiguration allied to the need of specialized instrumentation are the main reasons for the lack of biosensing technologies in the market and the sustained dominance of ELISA assays in standard molecular testing. Using the AptaShield DNA aptamer, any rabbit IgG can be turned into a solid-phase (by immobilization of the RAH or AptaBody DNA aptamer) or solution-phase biosensor overcoming current issues associated with most biosensors and immunoassays (such as ELISA).^13,21^ With the AptaShield biosensor technology, signal transduction takes place in bulk solution without the need for chemical immobilization to a biosensor surface.

Furthermore, rabbit antibodies are considered to have a higher affinity and specificity than other antibodies, such as mouse. They are highly specific and can bind to antigens at the picomolar range, while other mammalian antibodies recognize antigens at the nanomolar range.^22^ Due to these advantages, rabbit antibodies are becoming an asset in many clinical diagnostic assays.

In conclusion, the significant added value of the AptaShield biosensor is the capacity for universal reconfiguration, fast (less than 5 min), and high-throughput quantitative detection of different analytes in different complex biological matrices. The use of common laboratory instrumentation for the AptaShield biosensor expands its potential applications, greatly increasing the versatility and affordability.

## Supporting Information Available

Materials and methods, aptamer sequence, and physical properties (PDF)
