# Supplementary material for: AptaShield: A Universal Signal-Transduction System for Fast and High-Throughput Optical Molecular Biosensing
Source: ACS Sens. 2024 Apr 15;9(4):1756–62. doi: 10.1021/acssensors.3c02762 (PMC11059090; doi:10.1021/acssensors.3c02762)
Supplement: Supplementary file 1 — se3c02762_si_001.pdf [file se3c02762_si_001.pdf]

Supplementary Information

AptaShield: A Universal Signal-Transduction System for Fast and High-Throughput Optical Molecular Biosensing

Miguel António Dias Neves[1,2,*], and Inês Mendes Pinto[1,2,*]

[1]Institute for Research and Innovation in Health (i3S), University of Porto, 4200-135 Porto, Portugal

[2]Molecular and Analytical Medicine Laboratory, Department of Biomedicine, Faculty of Medicine, University of Porto, 4200-319 Porto, Portugal

*Correspondence should be addressed to:

Miguel António Dias Neves (migueln@i3s.up.pt) and Inês Mendes Pinto (ines.pinto@i3s.up.pt)

Materials and Methods

Unless otherwise specified, all reagents were purchased from Sigma-Aldrich and used as received. Furthermore, all buffers and aqueous solutions were prepared using ultrapure distilled deionized water (ddH_2O) with a measured resistivity ≥18.0 MΩ·cm. The AptaShield DNA aptamer was synthesized by Integrated DNA Technologies (Leuven, Belgium).

Isothermal Titration Calorimetry

For isothermal titration calorimetry (ITC) experiments, the AptaShield DNA aptamer and IgG samples were exchanged into PBS buffer (10 mM phosphate buffer, 2.7 mM KCl and 137 mM NaCl, pH 7.4 at 25 °C.). ITC experiments were performed using a GE VP-ITC instrument. Data were fit to a one set of sites binding model using Origin 7 software. Samples were degassed prior to analysis using the GE ThermoVac unit. All experiments were acquired at 25°C and consisted of 29 successive injections 10 µL injections spaced every 400 s, where the first injection was 2 µL to account for diffusion from the syringe into the cell. Binding experiments were corrected for the heat of dilution of the titrant. AptaShield DNA aptamer titration experiments were conducted by titrating 80 µM of aptamer into 2.5 µM of IgG (rabbit anti-human Fc specific, purified rabbit IgG, or mouse anti-human IgG), with the AptaShield in the syringe and the IgG in the sample cell.

Rabbit IgG Fluorescence Labelling

Monoclonal rabbit anti-human IgG Fc-specific antibody was first exchanged into Bicarbonate reaction Buffer (2 mM NaHCO3, 100 mM NaCl, pH 8.3 at 25°C). The rabbit IgG was reacted with AlexaFluor 594 NHS Ester (ThermoFischer Scientific) at a 10x molar ratio in reaction buffer for 2 hours at RT. The reaction was stopped with ethanolamine at a 2x molar ratio of AlexaFluor 594 NHS in reaction buffer. The labelled rabbit IgG was then washed with PBS buffer (10 mM phosphate buffer, 2.7 mM KCl and 137 mM NaCl, pH 7.4 at 25 °C) using Millipore Amicon 20k centrifugal filters until three clear filtrates were achieved.

Fluorescence Labelling Efficiency

Fluorescence labelling efficiency was evaluated by UV-VIS spectrophotometry using a Nanodrop 1000 Spectrophotometer (Thermo Scientific). The UV-VIS spectra of a sample of ALEXA-594 rabbit IgG were acquired (230 – 750 nm) using the appropriate molar extinction coefficients (Table S2) the concentration of IgG and ALEXA-594 were determined. The same procedure was used to confirm the fluorescence labelling of the AptaShield DNA aptamer with ALEXA-647 (Extinction Coefficients Table S1 and S2).

Titration of AptaShield DNA aptamer into Rabbit anti-Human IgG (and vice-versa) monitored by FRET

Different concentrations of ALEXA 647-AptaShield DNA aptamer or ALEXA 594 – rabbit anti-human IgG Fc-specific were titrated into 200 nM ALEXA 594 – rabbit anti-human IgG Fc-specific or 200 nM ALEXA 647 – AptaShield DNA aptamer respectively. The fluorescence emission spectra (Ex 550 nm) from 600 to 700 nm or just the emission intensities at 620 and 670 nm were acquired, using a Molecular Devices SpectraMax iD3. Binding curves related to AptaShield DNA aptamer/IgG titrations were produced by plotting the intensity ratios of 670/620 nm or 620/670 nm versus concentration of the titrant. Of note, the fluorescence intensity was background subtracted against the fluorescence signal obtained from the corresponding concentrations of 647 labelled AptaShield DNA aptamer. The apparent dissociation constants ($K_{d\ Apparent}$) were determined by fitting the binding curves to a one set of sites binding model using GraphPad Prism 8 software.

AptaShield Biosensor

Three proof-of-concept AptaShield biosensors were produced by mixing 150/50 nM, 300/50 nM and 600/50 nM of ALEXA 647 labelled AptaShield DNA aptamer and ALEXA 594 labelled Rabbit anti-human Fc-specific IgG (594-RAH). Purified human IgG Fc fragment (Abcam) was used as the analyte. To a 384 well plate format containing 18.75 µL of a specific AptaShield biosensor, 6.25 µL of purified human Fc spiked

in either PBS, bovine urine (European Union Reference Material, ERM) or horse blood serum (Sigma Aldrich) was added. The fluorescence intensity was determined (Ex 550 nm, Em_{donor} 620 nm (I_D), $Em_{acceptor}$ 670 nm (I_A)) using a Molecular Devices SpectraMax iD3 and background subtracted against the signal from the corresponding concentration of ALEXA 647 labelled AptaShield DNA aptamer. FRET concentration response curves were constructed by plotting (I_D/I_A) versus concentration of analyte (purified human Fc). The apparent dissociation constants (Kd apparent) were determined by plotting and fitting $FRET^{-1}(I_A/I_D)$ concentration response curves to a one site binding model using GraphPad Prism 8 software. The concentration limit of detection (CLoD) was determined from the residual standard deviation of the from the least squares regression of the linear region of the $FRET^{-1}(I_A/I_D)$ concentration response curves.[1–3]

Enzyme-Linked Immunosorbent Assay (ELISA)

A 96-well plate (Nunc MaxiSorp) was coated with rabbit anti-human IgG Fc specific capture antibody (Sigma Aldrich) by incubation of 50 µL of 10µg/mL of antibody in immobilization buffer (0.1 M sodium carbonate/bicarbonate, 0.1 M NaCl, pH = 9.6) overnight at 4°C. The plate was washed 3x with TBS-T (0.01 M TRIS, 0.1 M NaCl, 0.05 % TWEEN-20, pH=7.4) and then blocked by incubation with 200 µL of 3% skim milk (ED Millipore) in TBS-T for 1hr at RT. The plate was washed 3x with TBS-T. For purified human Fc (Abcam) binding experiments, 100 µL of spiked PBS or ¼ diluted horse serum samples were incubated overnight at 4°C. The plate was then washed 3x with TBS-T followed by incubation of 100 µL of anti-human Fc-HRP labelled antibody diluted at a 1:25 000 ratio. The plate was washed 3x with TBS-T and incubated for 15 min with 100 µL 1x TMB substrate solution (Invitrogen)and then stopped with 100 µL TMB stop solution (Sigma Aldrich). The absorbance of the plate was read at 450 nm and background subtracted against the signal from wells that were incubated with analyte free PBS or ¼ diluted horse serum. The dissociation constant and concentration limit of detection were determined as described above.

Table S1: Sequence and physical properties of the AptaShield DNA aptamer

AptaShield DNA aptamer sequence	5' – Alexa647 – CCAACCACACATCCTTCCATCGACATGGACCCACCGTTCC – 3'
Extinction Coefficient (260 nm)	394100 L/(mol·cm)
Molecular Weight	13031 g/mol

Table S2: Extinction coefficients of different reagents

Rabbit Anti-Human IgG Extinction Coefficient (280 nm)	210000 L/(mol·cm)
Alexa 647 Extinction Coefficient (650 nm)	270000 L/(mol·cm)
Alexa 594 Extinction Coefficient (590 nm)	92000 L/(mol·cm)

Table S3: ITC derived thermodynamic and binding parameters of AptaShield DNA aptamer binding to IgGs.

**Parameter**	**Rabbit Anti-Human IgG**	**Purified Whole Rabbit IgGs**	**Mouse Anti-Human IgG**
**N**	1.62 ± 0.07	1.66 ± 0.09	
**K_d**	224 ± 92 nM	890 ± 657 nM	No Binding
**ΔH**	-16.2 ± 4.2 kcal mol^{-1}	-4.0 ± 2.4 kcal mol^{-1}	Detected
**-TΔS**	7.2 ± 4.2 kcal mol^{-1}	-4.4 ± 2.8 kcal mol^{-1}	

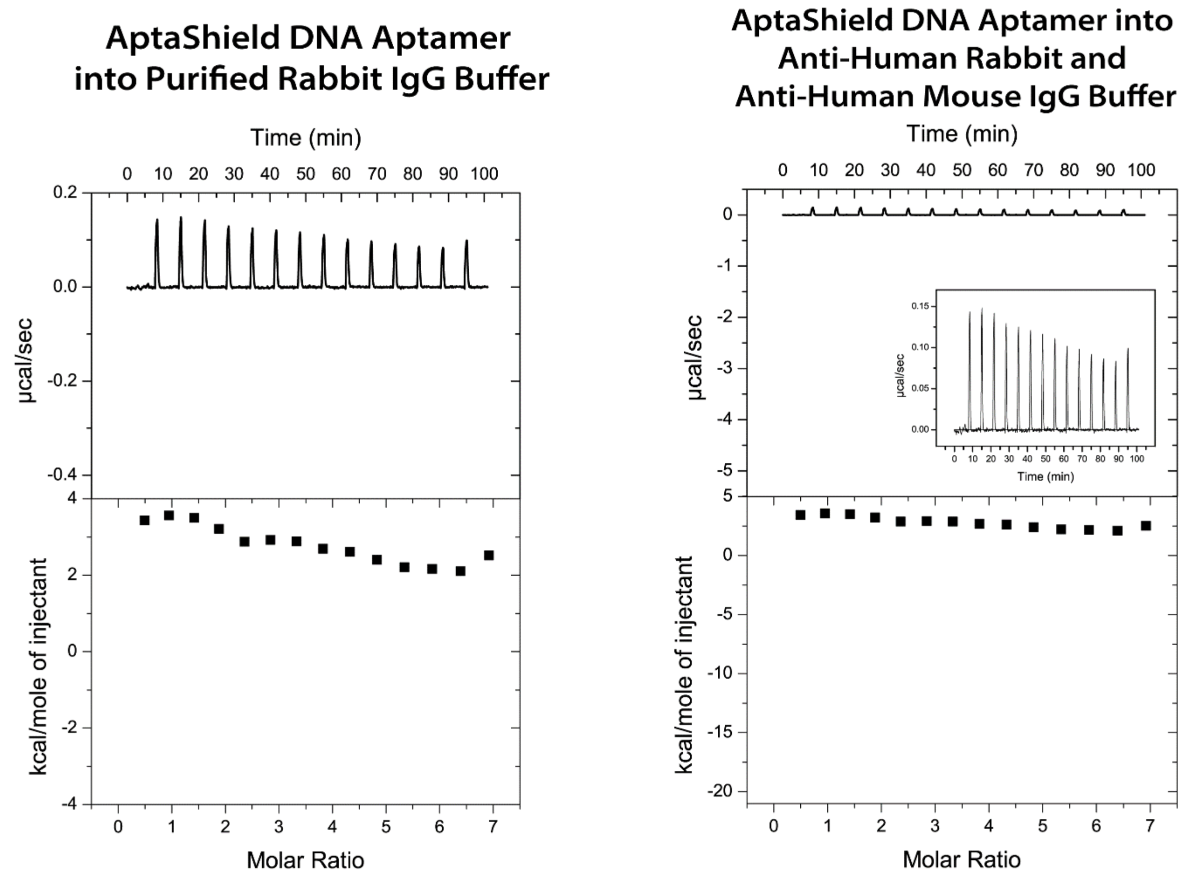

Figure S1: Isothermal titration calorimetry titrations to determine heats of dilution of the titrant.

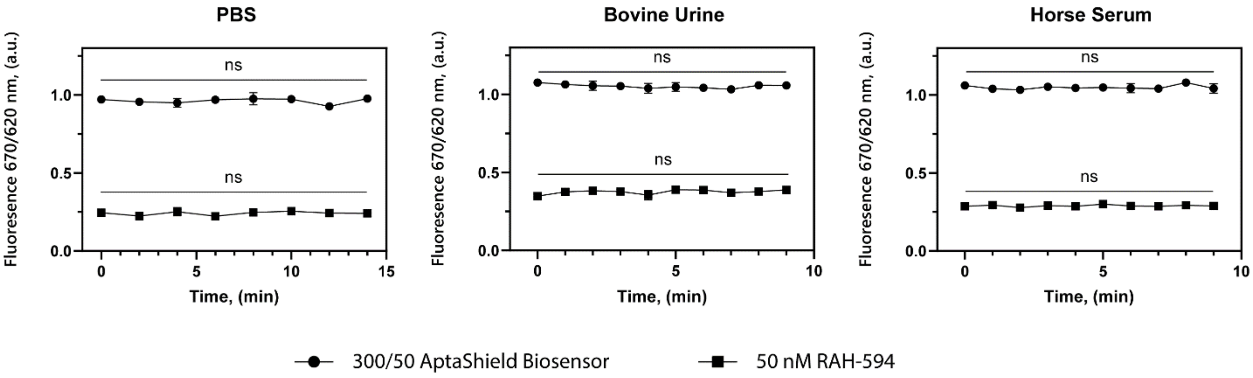

Figure S2: Repeated measurements over time of the fluorescence ratio (670/620 nm) of 300/50 AptaShield biosensor (uncorrected for AptaShield DNA aptamer-647 signal) and 50 nM RAH-594 in PBS, ¼ diluted bovine urine and ¼ diluted horse blood serum. The data shows that the ALEXA 594 on the RAH does not experience significant photobleaching and the fluorescence signal is stable over repeated measurements during a 15 minute time frame in PBS and a 10 minute time frame in urine and blood serum. The data is presented as mean ± SD with n=3.

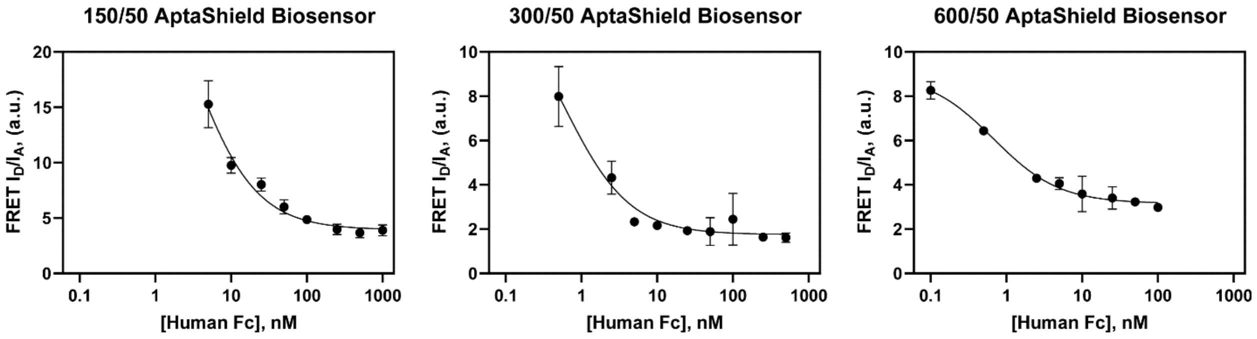

Figure S3: FRET (I_D/I_A) response curves of 150/50, 300/50 and 600/50 AptaShield biosensors to different concentrations of human Fc fragment in PBS.

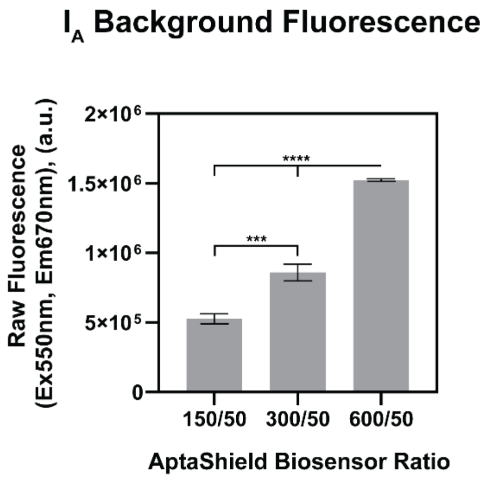

Figure S4: I_A Background fluorescence that must be subtracted from the corresponding 150/50, 300/50 and 600/50 AptaShield biosensors prior to calculating FRET (I_D/I_A).

References:

(1) Neves, M. A. D.; Blaszykowski, C.; Bokhari, S.; Thompson, M. Ultra-High Frequency Piezoelectric Aptasensor for the Label-Free Detection of Cocaine. *Biosens Bioelectron* **2015**, *72*, 383–392. https://doi.org/10.1016/j.bios.2015.05.038.

(2) US Food and Drug Administration. *Guidance for Industry: Q2B Validation of Analytical Procedures: Methodology*; Rockville, MD, 1996.

(3) Miller, J. N.; Miller, J. C. *Statistics and Chemometrics for Analytical Chemistry*, Sixth Edit.; Pearson Education Limited: Harlow, UK, 2010; Vol. 46.
